# Critical assessment in the age of AI: challenges and opportunities for computational biology

**DOI:** 10.1093/bioadv/vbag290

**Published:** 2026-10-16

**Authors:** Russell Schwartz, Xuegong Zhang, Jian Ma, Yana Bromberg, Robert F Murphy, Lawrence E Hunter, Laxmi Parida, Julio Saez-Rodriguez, Gustavo Stolovitzky, Mihai Pop, Shoba Ranganathan, Donna K Slonim, Terry Gaasterland

**Affiliations:** Ray & Stephanie Lane Computational Biology Department, Carnegie Mellon University, Pittsburgh, PA 15213, United States; Department of Biological Sciences, Carnegie Mellon University, Pittsburgh, PA 15213, United States; Department of Automation/BNRIST and School of Life Sciences, Tsinghua University, Beijing 100084, China; Ray & Stephanie Lane Computational Biology Department, Carnegie Mellon University, Pittsburgh, PA 15213, United States; Department of Biology and Department of Computer Science, Emory University, Atlanta, GA 30322, United States; Ray & Stephanie Lane Computational Biology Department, Carnegie Mellon University, Pittsburgh, PA 15213, United States; Department of Pediatrics, University of Chicago, Chicago, IL 60637, United States; IBM Research, Yorktown Heights, NY 10598, United States; European Molecular Biology Laboratory, European Bioinformatics Institute (EMBL-EBI), Hinxton, Cambridgeshire, United Kingdom; Faculty of Medicine, and Heidelberg University Hospital, Institute for Computational Biomedicine, Heidelberg University, Heidelberg, Germany; Department of Pathology, NYU Grossman School of Engineering, New York, NY 10016, United States; Department of Computer Science, University of Maryland, College Park, MD 20742, United States; Applied Biosciences, Macquarie University, Sydney, NSW 2109, Australia; Department of Computer Science, Tufts University, Medford, MA 02155, United States; Bioinformatics & Systems Biology Program, UC San Diego, La Jolla, CA 92093, United States

## Abstract

**Motivation:**

Effective computational tool development depends on our ability to measure progress with rigorous benchmarking, allowing developers to showcase advances and users to select the best of the available options. Computational biology has developed a robust infrastructure of benchmarking standards, tools/resources, and best practices to allow for rigorous and unbiased assessment. This infrastructure, however, faces growing challenges as modern artificial intelligence (AI) methods move to the forefront of computational analysis. The field has imperfectly adapted to issues problematic for benchmarking AI methods, including data entanglement, data leakage, problem-specific performance, limited theoretical guarantees, elusive ground-truth, opaque and often closed models that make reproducibility of results difficult, and dependence on impermanent data sources.

**Results:**

In this paper, we consider how the field should tackle benchmarking in the AI era. We revisit why benchmarking matters and its history in computational biology, examine emerging AI-specific challenges, and outline practical paths forward. We argue that benchmarks should be treated as long-lived community infrastructure rather than one-off artifacts bundled with individual papers. We envision a future of critical assessment i.e. dynamic and responsive to evolving use cases, sustaining progress for advancing science in the age of AI.

**Availability and implementation:**

N/A

## 1 Introduction

Benchmarking, the process of evaluating a method’s accuracy, computational efficiency, and other performance characteristics using real or synthetic datasets, plays a crucial role in the scientific enterprise (Stapenhurst 2017). Benchmarking depends on one or more benchmarks, combinations of datasets and evaluation protocols used to assess performance of a class of methods. Benchmarking is a key part of critical assessment, which involves evaluation of performance on benchmarks together with critical evaluation of the benchmark itself and the mechanisms for conducting it in an effort to derive rigorous and objective comparisons. Benchmarking has a long history in computational tool development, leading to a general understanding of best practices across computational domains. For more background on benchmarking, see, e.g. (Mangul et al. 2019, Weber et al. 2019, Brooks et al. 2024, Mallona et al. 2025) which provide earlier reviews of major issues in benchmarking computational tools for biological data, historical challenges to accomplishing this, guidelines for success, and practices by which this may be carried out. Sound benchmarking allows developers to assess proposed new methods and ensure that they are fairly compared to the state-of-the-art. It allows users to select the methods that are best suited to a given task. It gives reviewers and editors of publications the ability to assess the degree, if any, to which a new method improves on the prior art and thus partially judge the significance of its contribution to the scientific literature. Funding agencies benefit when applicants, demonstrating methodological rigor and realistic awareness of assessment criteria, clearly articulate the specific benchmarks against which their proposed methods will be evaluated. Benchmarking is ultimately a key part of a social contract by which communities must agree not only on metrics and datasets, but also on norms for disclosure and access to models and data, and on how to interpret performance claims under uncertainty. Note that we are not using the term ‘metric’ here in its strict mathematical sense but rather as the standard term of art for an evaluation measure in the machine learning (ML) literature. See Table 1 for a list of key terminology and brief definitions to assist the reader in following discussion of this topic.

**Table 1:** Key concepts useful for following this paper.^a^

Term	Definition
**AI**	A subdiscipline of computer science focused on mimicking human reasoning through computational models
**Benchmark**	A combination of datasets and evaluation protocols used to assess performance of a class of methods
**Benchmarking**	The process of measuring method accuracy and efficiency in evaluating real or realistic data sets
**Challenge**	A competition-based model for benchmarking using data with a known but hidden ground truth
**Critical assessment**	An extension of benchmarking with critical evaluation of the benchmark itself and the mechanisms for conducting
**Cross validation**	Controlling for overfitting by ensuring that ML models are tested on data independent from that used to train them
**Data leakage**	Unknowingly validating an ML method on data used in its training leading to biased assessment
**Deep learning**	An application of neural networks distinguished by its use of large networks with many layers
**FM**	A deep learning model trained on a broad, general data set allowing it to be specialized to diverse applications
**LLM**	A deep learning model for text or text-like streams of structured data
**Leaderboard effect**	Excessive focus of a field on success on benchmarks that imperfectly represent actual practice
**ML**	A subdiscipline of computer science focused on training explanatory models from data
**Neural network**	A class of ML model inspired by biological neurons
**Overfitting**	Poor generalizability of an ML model resulting from an overly complex model and/or insufficient training data

a This table lists some important concepts for the subject matter of this paper and provides brief definitions.

Thе need for benchmarking has proven particularly important in the field of computational biology as the life sciences have become critically dependent on computational tools for modeling, data analysis, and prediction (Markowetz 2017). This is an important need given concerns about reproducibility and replicability (National Academies of Sciences, Engineering, and Medicine et al., 2019) that cannot be addressed without trustworthy data analyses. Reliable benchmarking is especially necessary in interdisciplinary areas like computational biology (Hua and Zhang 2019), where key stakeholders may not themselves be experts in all sides of the discipline (here, wet-lab or compute aspects) but nevertheless must make high-stakes decisions about which tools to use, allow in a publication, or support with funding. It has become widely accepted in computational biology that publishable work must be open, properly evaluated for data leakage, and assessed in comparison to leading alternative methods to show a clear advance on prior art. In some disciplines, standard benchmark datasets and measures of success have been formalized to allow fair comparison across available tools for a given task.

However, benchmark datasets developed in conjunction with new analytical methods can lead to bias. The publication of such datasets on their own without biological or analytical innovations can be challenging; improved resources may not be reviewed as favorably as the discoveries they can enable. Such contradictions have led at least two domain-specific journals to create new benchmarking article types and collections, encouraging benchmarking submissions that follow rigorous standards (Peters et al. 2018, Robinson and Vitek 2019). The frequent use even of publicly available and acknowledged benchmark datasets can itself become a problem as well, as developers learn to ‘tune to the test’ (Norel et al. 2011) and optimize their methods for the standard benchmarks in ways that may generalize poorly to real applications.

In computational biology, a particularly successful and influential type of benchmarking was developed to prevent this problem: the challenge model, where test data with a known but hidden ground truth form the basis for a competition between tool developers who are evaluated on a shared set of benchmarks with agreed-upon evaluation criteria to provide a fair and unbiased assessment. The challenge model was first implemented by the U. S. National Institute of Standards and Technology Text REtrieval Conference (Harman 1992). It came to prominence in computational biology with the Critical Assessment of Structure Prediction (CASP; Shortle 1995, Zhang et al. 2026), a challenge focused on predicting protein three-dimensional structures from sequence data, using as the gold standard proteins whose structures had been recently solved experimentally but could be temporarily kept secret. CASP came to be widely accepted by the structure prediction community and is credited with providing a major impetus for progress in the discipline by making it possible to identify methods that made real practical improvements in the state-of-the-art. CASP facilitated dramatic improvements in structure prediction, such as when it marked the Rosetta method (Das and Baker 2008) as far ahead of competitors in de novo prediction and then, eventually, heralded the replacement of Rosetta-like methods with AlphaFold2 (Jumper et al. 2021) and its deep learning progeny. The success of CASP led to many similar challenges such as the Grand Challenges in medical image analysis (https://grand-challenge.org/), the Genetic Analysis Workshops that for 20 years conducted similar challenges for the statistical genetics community (MacCluer et al. 1984, Witte et al. 2007), the Dialogue for Reverse Engineering Assessment and Methods (DREAM) Challenges (Saez-Rodriguez et al. 2016, Meyer and Saez-Rodriguez 2021) that have covered many tasks in genomic data analysis and translational biomedicine, and the various Critical Assessment challenges (Critical Assessment of Genome Interpretation (Hoskins et al. 2017), Critical Assessment of Function Annotation (Zhou et al. 2019), Critical Assessment of Massive Data Analysis (Labaj et al. 2023), Critical Assessment of Protein Interactions (Wodak et al. 2023), Critical Assessment of Hit-Finding Experiments (CACHE; Ackloo et al. 2022), Critical Assessment of Metagenome Interpretation (Sczyrba et al. 2017), etc. as well as numerous more specialized one-off challenges. Table 2 provides a reference to a few of the important challenges used for critical assessment in computational biology.

**Table 2:** Some important benchmarking challenges in computational biology.^a^

Challenge	Methods assessed	Citation(s) and link(s)
**CASP**	Predicting molecular (primarily protein) structures	(Shortle 1995, Zhang et al. 2026)
**Grand Challenges in Medical Image Analysis**	Various applications of machine learning in medical image analysis	https://grand-challenge.org/
**GAW)**	GWAS	https://grand-challenge.org/
**DREAM Challenges**	Changing topics covering a diverse collection of current hard problems in computational biology	https://dreamchallenges.org/ (Meyer and Saez-Rodriguez 2021)
**CAGI**	Interpreting functional impact of genetic variations	https://genomeinterpretation.org/ (Hoskins et al. 2017)
**CAFA**	Predicting biological function from sequence and/or structure	https://biofunctionprediction.org/cafa/ (Zhou et al. 2019)
**CAMDA**	Diverse topics in analysis of big data in biology	(Labaj et al. 2023)
**CAPRI**	Predicting protein interactions, complexes, and assemblies	(Wodak et al. 2023)
**CACHE**	Small-molecule binding to disease association targets	https://cache-challenge.org/ (Ackloo et al. 2022)
**CAMI**	Interpreting the output of metagenomic sequencing experiments	https://cami-challenge.org/—(Meyer et al. 2022, Sczyrba et al. 2017)

a The table provides examples of influential challenges, providing the name, a brief description, and references or links to information about the challenge.

The challenge model and similar best practices have served computational biology well for decades but, as we show in Section 2, they are increasingly proving insufficient for a new landscape of method development in the era of big-data artificial intelligence (AI) and other data-intensive ML methods. Here, we will use the term AI to refer collectively to these data-intensive ML methods, including the deep learning-based methods underlying much modern AI, although we recognize that not all AI is ML and not all ML requires large amounts of data. AI methods trained on vast and heterogeneous data sets, which might include newer transformer-based foundation models (FMs) or prior deep neural network approaches, face a much harder challenge compared to classic algorithmic and statistical methods in finding truly independent test sets that do not unfairly inflate assessments of some competitors through unintentional data leakage (Kapoor and Narayanan 2023). Opaque training data and closed, application programming interface (API)-only model access make it difficult to evaluate competitors fairly and can make it at all but impossible for methods built on general FMs to be evaluated on a common platform with more traditional methods that do not present the same risks of undetected data leakage. Issues of hyperparameter sensitivity, complex multimodal data sources, and agentic workflows further complicate the problem of providing a fair, objective, and uniform test of progress that will allow the community to find what are truly the best methods for any given space of applications.

This article is intended primarily for readers who may need to assess computational biology methods, their own or third-party, or to critically evaluate the results of such assessments. In the remainder of this paper, we evaluate the challenges and propose potential solutions to the problem of benchmarking computational biology methods in the age of AI. We next summarize some of the particular difficulties of adapting past practices to current AI models. We then propose potential approaches for addressing these challenges going forward. Finally, we conclude with some consideration of unsolved problems, future opportunities, and a call to action for the research community.

## 2 Challenges of benchmarking for AI models

### 2.1 The challenges of classic benchmarking in an era of AI

Evaluation of computational methods traditionally relies on two key assumptions: (i) that there is a ground truth known through experimental evidence or theory and (ii) that this ground truth or ‘gold standard’ data fairly represent the real biological systems of interest, independent of design or tuning. The rise of powerful AI models promises to solve increasingly sophisticated biological problems, but as we push AI into more nuanced biological domains, these two assumptions are increasingly untenable. This creates significant challenges for fair and meaningful evaluation and increases the risk of false discoveries that are not easily detectable.

#### 2.1.1 Data leakage and overfitting

Classic benchmarking assumes that method design is independent of the test data. In ML models, performance is typically highly dependent on the data on which a model is trained. Assuring comparable independence can thus be more problematic if there is unrecognized reuse between training and benchmarking data, which has been termed data leakage. Minimizing this leakage and requiring that only well-defined training data are used for model fitting reduces this problem (Kapoor and Narayanan 2023), although it can be challenging to choose a stratification of the data for cross validation that will prevent similar data points from appearing in train and test splits. Model performance is also typically sensitive to choices in architecture and hyperparameters, which may be tuned using training and validation data through a process i.e. often opaque and poorly documented (and thus hard to reproduce), creating a source of data leakage i.e. easy to produce even inadvertently and difficult to detect *post hoc*. For example, pioneering transformer-based models pretrained on massive single-cell RNA-seq data show great promise as early prototypes of AI virtual cell modeling (Cui et al., 2024; Hao et al. 2024b; Theodoris et al. 2023), yet they reflect only narrow slices of a massive hyperparameter space (Bian et al. 2024; Hao et al. 2024a; Theodoris 2024). Assessment of overfitting can itself be surprisingly challenging, as overfitting to training data can paradoxically lead to better generalization to seemingly independent test data if data distributions are not carefully managed (Recht et al. 2019). These problems are amplified with extensively pre-trained AI models whose training data may be unknown to a developer retraining the model for a new task.

While independent test sets from community challenges help mitigate direct data leakage, the more subtle problem of the leaderboard effect arises: participants iteratively submit predictions to the test set, effectively performing implicit multiple testing across a vast combinatorial space of model variations. Similar to alpha inflation failures of early Genotype-phenotype correlation analysis and genome-wide association studies analyses, where the probability of finding a (false) positive association increases with every additional test performed (Chen et al. 2021), multiple re-testing of methods on benchmark data can lead to overfitting to the benchmark itself, inflating perceived performances without guaranteeing generalizability (Dwork et al. 2017). This problem can be mitigated by limiting the number of submissions to a leaderboard, as is typically done, e.g., in the DREAM challenges (Meyer and Saez-Rodriguez 2021). Techniques from privacy-preserving data analysis (Dwork et al. 2015) provide another potential mitigation strategy by which one might enable validation on a data set with provable limits on information leaks that could unfairly bias performance on future tests. These difficulties of data leakage and overfitting intensify, however, with advanced deep learning and transformer-based models, which operate in extremely high-dimensional hyperparameter spaces that create many opportunities for overfitting a model to its training data. For large pre-training models, it is also particularly difficult to ensure that benchmarking data is truly independent of the vast corpora used for pre-training, raising concerns about data leakage through experiments drawing on common systems, protocols, or experimental conditions in addition to direct reuse of shared data points.

#### 2.1.2 Biases in design of datasets

Reliable benchmarking demands experimental designs that reflect true biological variability, and ideally represent a random sample of the relevant space. However, the sample distributions of available datasets are often dictated by practical constraints—biological, technical, financial, ethical, and so on—rather than ideal experimental design. Consequently, a model’s performance is often idiosyncratic, tied to a specific problem, dataset, or technology platform. Moreover, biological data analysis often involves many expert-driven steps—such as pre-processing and clustering, not to mention the choice of what data to gather—that rely on subjective judgement. These practices can also mislead AI researchers into over-optimizing aspects of a model that may be peripheral to the actual biological discovery. Consider, e.g. a benchmark for detecting functional genomic variants that lacks examples of some important variant classes. If a model predicts that a genomic variant has functional impact while the benchmark suggests otherwise, then tuning the model to the benchmark may bury real biological findings that would be considered relevant if identified by an experiment (Bromberg et al. 2013, Mahlich et al. 2017, Zeng et al. 2021). Such choices shape conclusions profoundly but are often underreported in publications, leading to irreproducible results and misleading interpretations of AI contributions (Chen et al. 2024).

Another manifestation of bias in the biomedical data corpus is the disproportionate concentration of research on a relatively small subset of potential objects of study. Bias in data is not a problem exclusive to AI methods, but the use of AI and other data-intensive ML methods compounds the problem. Models trained on biased data learn to reflect those biases in ways that can be difficult to anticipate or analyze after the fact, a problem of growing concern as models become ever larger, more opaque, and more costly to train and audit. For example, biomedical research is highly skewed toward a small fraction of genes; an analysis of nearly 600 000 publications annotated to ∼20 000 human protein-coding genes revealed a scale-free, ‘Matthew effect’ distribution, in which a handful of proteins accumulate thousands of publications while hundreds have none (Carter et al. 2019). This imbalance persists despite genetic evidence linking many understudied genes to disease, suggesting that research attention is driven less by biological importance than by familiarity with particular systems and existing tools and funding incentives that result in a self-reinforcing ecosystem in which cumulative visibility begets further study.

Another example of this bias can be found in the protein structure world. Until the Protein Structure Initiative (2000–2015) pressed for emphasis on novelty in the world of protein structure determination, the vast majority of proteins in the PDB reflected specific field interests; over 70% of the newly determined structures were at least 95% sequence-similar to previously determined ones (Liu et al. 2007, Norvell and Berg 2007, Nair et al. 2009). If all prediction models were evaluated on this set of proteins, they would have only reflected less than three quarters of the currently known structural variety, which itself may be a small fraction of what is truly represented in nature. This is particularly problematic for protein families that only have a small set of PDB structures, e.g. insect odorant receptors, where AI models are not yet biologically accurate (Jabeen et al. 2024, Chidambara Thanu et al. 2025). When such uneven corpora are used to train AI, predictive performance is likely to be strongest for well-studied genes and weakest precisely where new discovery is most needed. This issue leads to systematic epistemic bias and cannot be solved by adding a few more datasets.

#### 2.1.3 Biases of evaluation metrics

The choice of evaluation metrics presents another layer of complexity (Baldi et al. 2000). Evaluation typically relies on measures comparing model outputs to human-defined ground truths. Therefore, state-of-the-art claims based on these metrics must be viewed cautiously, as such benchmarks assess only specific, constrained scenarios and may not translate to real-world biological utility. The organizers of the challenge models have continuous discussions about applicable metrics and their usefulness (Cokelaer et al. 2015, Kryshtafovych et al. 2021, Wodak et al. 2023, Critical Assessment of Genome Interpretation Consortium, 2024, Piovesan et al. 2024). These problems can also be mitigated, but not completely avoided, by the use of multiple independent metrics that may at least reflect different biases and assumptions [e.g. (Nielsen et al. 1996)] and make it more difficult to tune a method specifically to the benchmark’s evaluation metric, thus avoiding falling in the trap articulated in Goodhart’s law (Muller 2018). Strict definitions of applicable metrics of success, arguably, facilitate and disproportionately benefit advances in AI-based methods, which require continuously scored optimization end goals. Proper evaluation of many predictions may require predictions themselves to be standardized into a limited set of, e.g. ontology labels, which might limit expressiveness of experimental outputs, e.g. to the terms in the Rhea (Bansal et al. 2022), Enzyme Commission (Tipton and Boyce 2000), or Gene Ontology (Ashburner et al. 2000) nomenclatures. The choice of a suitable ontology thus also becomes part of the evaluation decision-making.

Second, many prediction tasks are built on unverified assumptions that may lead to circular reasoning and poorly chosen metrics may lead to tests of fidelity to the field’s assumptions rather than an actual biological ground truth. For instance, a common benchmark for multi-omic integration assumes that different omics data share an identical latent distribution, and that the ‘best’ model is the one that forces them to overlap most completely in the latent space (Ashuach et al. 2023, Heumos et al. 2023, Hu et al. 2024, Xiao et al. 2024) strong assumption that risks discarding important information uniquely inherent to different omic data types (Baião et al. 2025). Similarly, models that integrate single-cell and spatial transcriptomic data to translate between gene expression and spatial location, while highly useful, operate on a principle—that gene expression deterministically associates with location—that itself requires systematic validation (Biancalani et al. 2021, Hao et al. 2024c, Cao et al. 2025). Across many tasks, metrics and human-assigned ground truths reflect a bias for current understandings and for the outcomes researchers expect to find, which may unfairly penalize those methods that are most effective at challenging dominant paradigms and discovering new biology. This is not a problem unique to biology, but of learning-based methods in general.

Furthermore, standard performance metrics like mean squared error or correlations, may not reflect biological fidelity (Schäfer et al. 2026). They fail to account, e.g. for varying dynamic ranges of genes, their different sensitivities to sequencing technologies, and their different implications for cell fates. A numerically small error for a key regulator could be biologically more catastrophic than a large error for a stable housekeeping gene. In evaluating protein structure prediction, e.g. standard measures of accuracy such as root mean square deviation (RMSD) may be useless in evaluating structures that differ by position of a single hinge between rigid domains. Conventional measures of classifier performance, such as accuracy, precision/recall, or F-score may be highly misleading if test cases are unrepresentative of the real space of hard problems. Similarly, the silhouette score is frequently used for data integration, even though its simple model of clustering consistency is poorly suited for this purpose and may in fact penalize effective data integration that relies on differences in how complementary data sources cluster the same entities. Current evaluation frameworks are not yet sophisticated enough to capture such crucial distinctions.

#### 2.1.4 The elusive ground truth

Reliable benchmarking hinges on verifiable ground truth. While this might seem straightforward, it is a major bottleneck in real-world applications and perhaps especially in biology. Biological concepts like cell type or protein coding are often subjective and blurry at the boundaries, and the complexity is amplified by technical biases in data collection and historical artifacts in how data labels were defined, by whom, from what evidence, and for what purpose. Metrics that work on well-defined vision or language tasks may not fully capture biological realities, where tasks may be ill-defined and correct answers unknowable with certainty.

Consider the task of in-silico cellular perturbation prediction as an example. Large-scale perturbation datasets are invaluable [c.f. (Liberali et al. 2015)], but they are imperfect as ground truth due to multiple limitations. Cellular responses to a perturbation in experiments are a mixture of true perturbation effects and artifacts from the perturbation technology itself, such as DNA damage repair triggered by CRISPR-based gene perturbation. The destructive nature of many measurement technologies may make it virtually impossible to extract several channels of information about the same entity (e.g. to measure full transcriptomes and other omics data from the same cell both before and after perturbation) so predictions can only be evaluated at the population level. Many experiments can only be performed in cell cultures or other laboratory environments, which may differ from cells’ real responses in the complex physiology of an *in vivo* microenvironment.

Even a seemingly straightforward application like protein structure prediction—where X-ray crystallography, nuclear magnetic resonance, and now cryo-electron microscopy (cryo-EM) have provided what were treated as definitive ground truths for assessment—can yield subtle complications. A ligand-binding protein may have a set of plausible structures, flexible regions, or even completely disordered domains, so experimental data is only a snapshot of dynamic binding activity. Protein preparation for crystallography requires a highly unnatural environment for proteins or alterations of the native sequence that can yield subtle, or sometimes dramatic, changes in structure relative to native solution conditions (DePristo et al. 2004). Cryo-EM provides in principle structural information closer to that in the natural environment, yet even it may struggle when dealing with issues such as flexible, partially disordered, or polymorphic proteins (Punjani and Fleet 2021). Method developers and users of those methods both need to be familiar with the often-hidden assumptions and limitations of the technologies producing the data behind their benchmarks.

Furthermore, AI classification or prediction in biological studies often serves as an intermediate step toward new scientific insights and discoveries. Validating or refuting these findings may ultimately require additional prospective wet-lab experiments or even clinical trials, where biases in what is feasible create further problems. New experiments are often *ad hoc* and costly. For some findings, there may not exist any feasible way to validate them because of technical or ethical constraints. There is a pressing need for developing objective, standardized frameworks that incorporate feedback to assess and refine AI-driven discoveries, and that draw on both retrospective and prospectively generated data within technical and ethical limits.

Synthetic benchmark data can provide a partial answer to these problems, offering a source of ground truth in applications where it may otherwise be unknowable, such as in evolutionary biology. Synthetic data can be especially powerful in conjunction with real data. However, synthetic generation brings its own biases in producing data i.e. inherently tied to a specific model i.e. necessarily a highly simplified representation of the true biology. In addition, true data is often highly imbalanced, creating particular challenges to accurate benchmarking when synthetic data is disproportionately needed to augment negative examples typically lacking in published data resources.

#### 2.1.5 Gaps in theoretical foundations

All of the above challenges are amplified by gaps in our theoretical basis for making rigorous decisions in model design, training, and benchmarking tasks. AI methods are often highly heuristic in their application and dependent on empirical assessments of performance. This is in contrast to more traditional algorithmic methods, for which it is often possible to provide firm guarantees on measures of efficiency, complexity, accuracy, or data needs, and for which there are often few arbitrary decisions left to the method designer. This dependence on intuition and potentially arbitrary design choices has only become more true as deep-learning methods have displaced more straightforward ‘classic ML’ and led to highly complex models requiring numerous design decisions and hyperparameter settings. When these are set by intuition or often poorly documented empirical adjustments, rather than by agreed-upon algorithms backed by clear theoretical guidance, it creates an opportunity to amplify aforementioned problems of data leakage. This is not to demean the knowledge and expertise that can go into making these choices, but only to note that they create challenges for fair assessment.

The task of benchmarking itself is unlikely to be precisely defined in a formal theoretical sense, creating further opportunities for bias to enter into evaluation and in turn making it difficult to control for the sources of bias noted above. We are unaware of a sound theoretical foundation for rigorously defining the problem a benchmark is meant to solve, much less for providing guarantees on how well a given choice of benchmark solves that problem or designing algorithms for optimizing a benchmark to its formal problem statement.

### 2.2 The new AI: LLMs and foundation models

The promise of FMs offers a potential boon for making powerful AI available to those who lack the resources to train a model de novo as well as for extending data-hungry AI technologies to problems where data is too sparse to train sophisticated models or where zero-shot prediction is required (Moor et al. 2023). However, the large size of these models and the cost of training them raise their own special concerns for assessment (Saez-Rodriguez et al. 2026, Shu et al. 2026). This section considers a few of the particular challenges posed by assessment that these models raise.

The high cost of training these models and the large amount of data they require has generally meant that they are trained infrequently, often by private entities under proprietary conditions, creating inherent problems for transparency and reproducibility. These conditions can make straightforward benchmarking nearly impossible in practice. The training corpora used to produce these models are often proprietary themselves and guarded as trade secrets. This problem is amplified by the fact that new releases of these models occur regularly on updated training data i.e. too large to explicitly describe or back up for purposes of reproducibility. Extensive work in data preprocessing and hyperparameter selection is typically needed to get good performance from these models, and this process too may be treated as proprietary, providing hidden ways in which prior knowledge is subtly encoded into the design. This lack of transparency can propagate problems for downstream validation, as users of these models cannot be certain of the data on which they were trained or prevent data leakage.

A separate concern is that it is hard to evaluate the ability of FMs to accurately represent biological data even prior to any downstream data-driven analysis. For example, how good are the numeric vectors that replace amino acid sequences or structures when representing a protein? Protein language models (pLMs) vary in their ability to represent proteins, and while some metrics for representation evaluation have been proposed (Senoner et al. 2025, Prabakaran and Bromberg 2026), there is no generally accepted way to pinpoint the best pLM (or even if there exists a best amongst many possible representations) for every question that one can ask about proteins. In evaluating novel method performance on downstream tasks, the contribution of the final model cannot be disentangled from the input pLM’s abilities. Because downstream performance reflects the combined effects of both a pretrained model and the task-specific adaptation, it does not provide a direct measure of the pretrained model’s standalone quality independent of the added value of its task-specific fine-tuning. Independently benchmarking pLMs and FMs through their performance on zero-shot learning without fine tuning, can provide a partial answer. This prompts a deeper methodological question: what constitutes a sufficient and principled set of downstream evaluations to meaningfully assess a pLM or FM?

Similar issues arise in separating the contributions of a method from the data on which it is built. Even with full transparency on the architecture of an AI model and process by which it is trained, the high cost and proprietary nature of typical FMs means that there is no opportunity to assess how much of the improvement of a new model is due to changes in the model itself versus changes in its training data. Ablation testing strategies that have become commonplace, if not fully standardized, for addressing these issues with smaller-scale AI models (Meyes et al. 2019) are simply not feasible for large FMs. This further challenges good validation practice by making it difficult to protect against problems of data leakage that still plague validation even for older ML models.

These challenges to assessing the FMs’ training data can leak into assessment of downstream applications. Even an academic user seeking to follow best practices for openness and appropriate cross-validation may be limited by the lack of transparency if they seek to build on leading FMs. Truly open models, with open parameters and training data, provide a path forward but may raise difficult tradeoffs if they do not match the state-of-the-art performance of closed commercial models. This makes it difficult to assess the technical contribution of any new method building on an FM. Does improved performance of a specialized application of an FM relative to prior art reflect technical improvements in the FM, technical improvements in its specialization to the downstream task, better data underlying the FM, or better data available to it for retraining? Sorting out these possibilities is important to deciding whether a model offers innovations that should be picked up by others in future work. Yet the scientist retraining an FM for a new task may have only limited ability themselves to distinguish between these possibilities.

Bringing what were classically considered best practices for model assessment to this domain also raises new practical and ethical concerns (Ferdaus et al. 2026). Following accepted best practices for validation, with appropriate ablation testing and cross-validation on independent data sources, may be so costly that it is beyond the reach of the academic community. Questions of the carbon footprint of carrying out these tests further complicate the calculus of how to set responsible best practices (Liu and Yin 2024, Ferdaus et al. 2026).

### 2.3 Assessing intellectual merit in the era of AI

A more profound and critical issue for assessment is how we judge the promise of a new method beyond what is revealed by its quantitative performance. Is a model eking out performance improvements through conceptually trivial parameter tweaking and fine-tuning that may be made obsolete by the next data set, or are there big ideas behind it from which the field can learn? This philosophical question concerns the epistemological value of FMs (what they truly reveal about biology beyond predictive accuracy) and is a crucial practical question for such considerations as whether a new idea warrants publication, grant funding, or attention from the community. This is not to dismiss the importance of other bases by which a model may achieve superior performance to its predecessors, such as through investment in generating better data or through effective one-off engineering. Rather, it is to recognize as well that the intrinsic intellectual merit of a model is an important consideration in judging its significance and likely impact that may not be immediately apparent from its performance on a straightforward benchmark alone. In other words, models that achieve better fit to a target outcome possibly by virtue of their sheer number of parameters are not necessarily more illuminating from a conceptual standpoint or more likely to lead a path to further advances (Fernandez Slezak et al. 2010).

Classic algorithmic methods were frequently judged by a quasi-aesthetic sense of the elegance of the contribution and the sense of surprise it evoked (Sinclair 2011). The neural network models currently dominating AI are frequently distinguished from one another by engineering design choices as opposed to truly new theoretical ideas. Engineering has its own aesthetic (Schummer et al. 2009), but one difficult for engineers themselves to articulate and even more difficult to balance against the hard numbers one seeks in a quantitative assessment. However, when improved performance of a new model may itself be a product of improvements in the data available to it or in an FM it uses as a base, it becomes particularly important to be able to assess what new value the model adds to the field strictly on its own intellectual merits.

The history of de novo protein folding prediction provides a useful case study. Here, early computational work using classic physics-based methods provided useful conceptual insights into protein folding processes [e.g. (Sali et al. 1994)]. Neural networks helped the analysis of protein evolutionary profiles to predict folding of sequences into secondary structure (Rost and Sander 1993) but true, reasonably accurate folding prediction was only possible for specialized classes of proteins with highly restricted structure spaces, such as coiled coils (Lupas et al. 1991). The Rosetta method (Das and Baker 2008) proved groundbreaking, both in a conceptually new approach combining statistical and physical methods and in a striking improvement in performance in CASP assessments relative to anything that came before (Simons et al. 1999). In 2012, using amino acid co-variation computed from extremely large multiple sequence alignments (MSAs), Marks et al. (2012) were able to reproduce protein structures for a set of very common proteins. Four years later, Golkov et al. (2016) showed that analyzing MSAs with Convolutional Neural Nets, instead of hand-crafted features, allowed for superb performance. AlphaFold2 (Jumper et al. 2021, Chang and Perez 2025) represented a major leap in empirical performance, replacing earlier learning strategies with transformer models able to train on the large repositories of known structures and MSAs the structural biology community had been assembling for decades (Sussman et al. 1998, Berman et al. 2000). While AlphaFold2 did not completely solve the protein folding problem—e.g. it continued to struggle with predicting structures of fold-switching proteins (Chakravarty et al. 2024)—it yielded a dramatic improvement over the prior art. Here, a conceptual leap in the design of the AlphaFold2 transformer model was followed by a large investment in compute resources needed to train and test the model and the two together ultimately led to a massive improvement in quality of prediction, assessed with quantitative metrics. A similar example can be seen in dramatic advances in language recognition technologies that were fueled by major investments in data generation that could pay dividends once the foundational ML methods had been developed (Alspector and Dietterich 2020).

Such histories raise the question of whether there was anything about the conceptual leap itself from which the community could have concluded in advance that the large investment needed to capitalize on it would ultimately be justified. There are case studies of traditional science funders balking at outsider ideas or the investments needed to capitalize on them, only for non-traditional sources to step in and prove their value. Celera Genomics provided a seminal example of private investment driving a revolution in genome sequencing by enabling a computationally intensive shotgun sequencing strategy, a key intellectual advance for the field at which traditional public funders had balked (Weber and Myers 1997, Venter et al. 2001, Cozzarelli 2003). Nevertheless, picking out good ideas before they are proven empirically is difficult but it is also crucial to advancing science when there are finite resources to spare to support scientific progress. That is only more true in an era of big AI models, when massive investments may be needed to train state-of-the-art models or to collect the data they require and hard decisions must be made regarding how much investment should be put into researching fundamentally new ideas versus capitalizing on the best current ones.

One of the reasons this kind of assessment is difficult in the era of modern AI is that, despite a great deal of work on the theoretical foundations of AI, we generally lack hard guarantees of performance that allow us to compare usefully between distinct deep learning models for a given task. Classic algorithms typically lent themselves to proofs of theoretical advantage over their predecessors. Even spaces where heuristic methods came to dominate, such as sequence searching and alignment, were backed up by extensive bodies of theory able to give guaranteed performance bounds under suitable assumptions. While AI-based methods are also backed by extensive theory, that theory has not led to comparable performance guarantees, and so the field must rely more heavily on empirical assessments with the problematic issues they raise about test generalizability and fairness.

Once again, history provides some models for how the field might need to evolve going forward. There is a long tradition of computational problems transitioning from being seen as AI to be solved by clever heuristics when the problems are poorly understood, moving to the domain of theory as the problem statements become better defined and yield to precise methods with provable performance guarantees, and finally coming to be seen as questions in computational system-building when both the problem and the theory behind solving them become settled. One example would be tasks in searching and planning that were once viewed as AI (Newell and Simon 1972), later replaced by rigorous graph theoretical formulations (Dijkstra 2022), and eventually became standard algorithms taught in introductory algorithms classes (Cormen et al. 2022) and routinely called from algorithm libraries. Another would be core problems in machine vision such as edge detection, that were once seen as a hard problem of classic AI (Roberts 1963), gradually yielded to more rigorous mathematical formulations (Marr and Hildreth 1980), and now have accepted methods (Canny 1986) that are standard parts of image analysis toolkits. We can at least conjecture that the kinds of problems typically solved by LLMs today may follow a similar trajectory of yielding to more rigorous problem formulations, which in turn lead to standard algorithms with provable guarantees that might look quite different from the currently prevailing neural network approaches and which will no longer be seen as AI.

## 3 Towards best practices for benchmarking AI

In this section, we recommend some best practices for benchmarking AI methods, as well as steps by which they can evolve with the field going forward. Our intention is to provide guidelines for those running benchmarks, users participating in benchmarks, and other stakeholders who need to make decisions based on their assessments. We also conclude with some consideration of areas in need of further research as future AI develops.

### 3.1 Defining formalized assessment criteria

Ensuring fair and rigorous benchmarking will require new standards for openness and reproducibility as well as mechanisms to ensure practitioners in the field are prepared to follow those standards. We recommend the following practices, summarized in Fig. 1:

**Figure 1:**
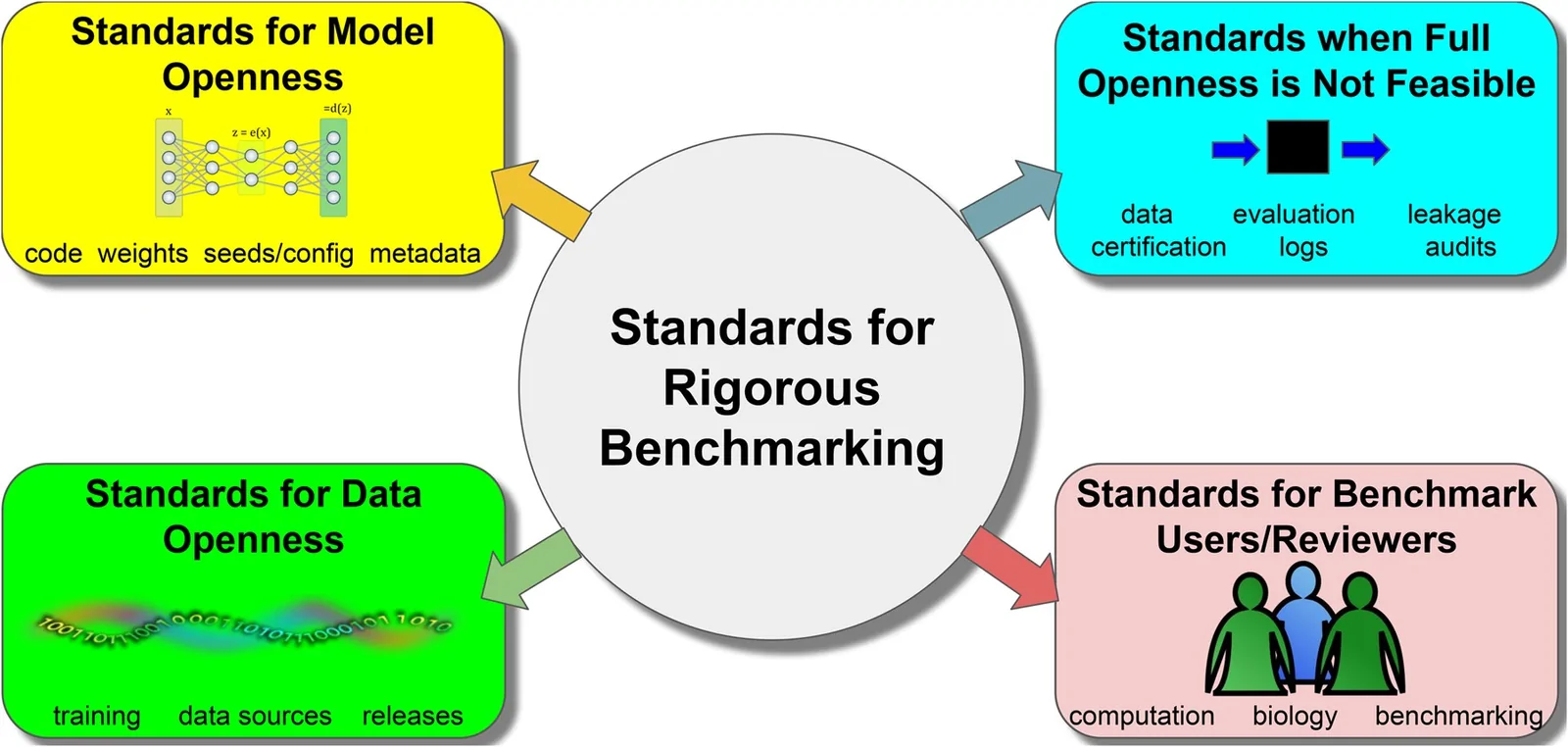
Summary of best practices for benchmarking AI models. The flowchart summarizes some of the principal recommendations of the paper for improving practices of benchmarking in the AI age. These include Standards for Model Openness (upper left) identifying model features necessary to rigorously assessing model training and performance; Standards when Full Openness is Not Feasible (upper right) for certifying data and evaluation protocols and auditing to ensure sufficient protection against data leakage during training and assessment; Standards for Data Openness (lower left) establishing what needs to be known about data used in AI models to allow for robust assessment; and Standards for Benchmark Users/Reviewers (lower right) to ensure model assessment is informed by the necessary expertise. Icons in the images are free for reuse and sourced from https://openclipart.org/detail/340524/artificial-neural-network (model openness), https://openclipart.org/detail/167103/binary-data-stream-2 (data openness), and https://openclipart.org/detail/35563/abstract-people (users/reviewers).

- Explicit standards for openness of models, including open code, weights, configs, seeds, and metadata describing how the model was trained and validated. The details of these standards may evolve with technology, but we believe that they should be explicitly stated and regularly revisited and updated. This would include specific standards in the instructions for authors of journals and conferences publishing in the field. Standards should similarly be part of grant evaluations as part of review criteria to ensure rigor and reproducibility. In addition, minimum reporting standards for AI-enabled biological analyses (model cards, data statements, and evaluation traceability) can reduce ambiguity about what was actually tested and under what conditions.
- For API-only models, providing training-data certification, evaluation logs, and leakage audits. We recognize that the field has moved to the point where it may no longer be possible to maintain expectations that models are fully open and available for independent review. New expectations are thus needed for how we ensure reproducibility under these circumstances.
- Explicit standards for openness of data used in model training, including identifying specific data sources and certified releases of living data sources at the moment of training. Just as fully open models may not always be possible, likewise for fully open training data, where use of dynamic data sets too large to archive individually and make available for independent review is becoming standard for large models. Time-stamped data snapshots and clear data cut policies dictating the criteria by which data are removed from a benchmark can help make evaluation comparable across model versions and across groups.
- Securing appropriately qualified reviewers. A necessary addition to the prior point is that review of materials that include computational biology tools, whether i.e. for publication, funding, awards, or other opportunities, must be done by reviewers competent to evaluate whether the above criteria have been followed. That is, those making decisions on deployment of computational tools should be expected to have competency in computational biology and benchmarking. It has long been recognized in the computational biology community that work that depends on computational analysis—i.e. nearly all work in biomedical science today—requires reviewers competent to review those analyses, and that extends to reviewers having sufficient understanding of benchmarking practice to make appropriate use of their results. AI itself may provide partial solutions, e.g. via human reviewers whose expertise is augmented by AI agents, although that raises other concerns.

### 3.2 Connecting assessment to data generation

A growing problem, alluded to several times above, is that modern AI models typically depend on training data that may itself be impossible to describe or evaluate directly. Much like the models, if data cannot be fully open then at a minimum the process that produced it should be fully documented, auditable, and in principle reproducible; data and metadata standards should be followed; and the data itself should be rigorously assessed. Meeting these expectations presents technical challenges to incorporating assessment directly into the data generation process, and even more so for community data generation efforts that depend on happenstance of data submissions rather than a central plan. It also requires a sea change in thinking about data generation, with rigorous thought to the computational uses of data incorporated into the process of data generation from conception through all stages of experimentation and validation. Steps in this direction are, e.g. being taken by initiatives such as the U.S. National Institutes of Health’s Bridge2AI Consortium (https://bridge2ai.org/). However, challenges remain in figuring out how to bridge the gap between often opaque industry practices and the need for openness in the scientific community as a whole.

Systematic efforts to gather more unbiased data sources can also help. A compelling example of how one might counter structural bias in data sources used in model training and benchmarking is Target 2035 (https://www.thesgc.org/target2035), which seeks to systematically generate high-quality chemical probes and large-scale protein–ligand datasets across the broader proteome (Carter et al. 2019). Its roadmap emphasizes standardized, openly accessible binding data at scale, including both positive and negative results, to enable robust and generalizable ML development (Edwards et al. 2025). By producing curated, AI-ready datasets and organizing benchmarking challenges with experimental validation, Target 2035 seeks not only to illuminate understudied proteins but also to reframe hit discovery as a more equitable, data-driven, and computationally accelerated enterprise. The first of what is expected to be a series of crowd-sourced challenges enabled by this effort has recently been launched through a collaboration between the DREAM and CACHE platforms, with support from the Structural Genomics Consortium (Sage Bionetworks).

Past assessment efforts have relied on expert evaluation and/or preprocessing of experimental datasets, despite no clear evidence that this improves the assessment process and significant concerns that models may not capture the inherent variation in such data. More deliberate strategies for planning effective generation of large data sets are also needed. As biomedical discovery has come to rely more on inference over complex multimodal data sources, there is a growing need for data generation itself to be recognized as a problem space over which one can optimize (Jin et al. 2005, Tarazona et al. 2020, Srivatsa and Schwartz 2024), a realization i.e. even more urgently needed for the large-scale data collection efforts powering sophisticated AI models today. Increasing moves towards self-driving and automated science [c.f. (Sparkes et al. 2010, Naik et al. 2016, Canty et al. 2025)], where AI training is tightly integrated with experimental design and data generation, offer both opportunities and further challenges. Approaches based on autonomous science and active learning, a class of ML in which model learning and data generation are interleaved to allow the model to select data points most informative to it, can potentially create a new paradigm for assessment i.e. independent of expert evaluation. Here, we start with the decision to make assessments based solely on the ability of models to predict novel experimental measurements for specified experimental conditions, rely on active-learning driven assessment cycles to query existing models to identify those combinations of conditions and predicted measurements in which the models themselves have the least confidence, design experiments to test those predictions and execute them using laboratory automation with minimal human intervention, and evaluate all models using the results. The benchmark data can then be released to allow model retraining, and another assessment cycle begun.

Progress on these problems is in turn likely to require reform in educational and administrative practices that go beyond computational biology. This includes ensuring that those planning or leading data collection efforts have the necessary knowledge and expertise in how those data will be used. It will also require new consideration for how funding is allocated to ensure that benchmarking efforts are not only supported but have the resources where needed for novel data generation.

### 3.3 Assessing innovation in the era of AI

Future benchmarking of AI methods will also be complicated by the need to disentangle the many factors that can contribute to improved empirical performance, so that we can separate assessment of the intrinsic merits of a model from the data and other models on which it depends. Future assessment of AI models will require robust ablation strategies to explore independently the influence of training data, fine details of model design, hyperparameter choices in model architecture and training, and genuinely new innovations in AI models or algorithms. The field will need to define how this is handled when domain-specific models depend on opaque FMs or proprietary training data. Analysis is further compounded by the difficulties of rigorous cross-validation noted above when models draw on vast and heterogeneous training data resources that may themselves be of uncertain provenance (Pop et al. 2025). Quantitative benchmarks cannot fully replace human judgement and an appreciation for the elegance of a solution (Sinclair 2011) when assessing genuinely new and innovative ideas with promise that may take time to develop.

### 3.4 Evaluating interpretability

A related question is interpretability of the outputs of complex models that seemingly perform well in benchmarking assessments (Linardatos 2020). The lack of interpretability has been a frequent criticism of neural-network-based AI models, although it has been tolerated as users have developed more confidence in assessing AI performances on natural language understanding or computer vision tasks. Interpretability is arguably particularly important on computational biology tasks (Chen et al. 2024), where users are often not experts in AI methods but want to understand their results and evaluate them according to their own professional judgement. Interpretability can help us to assess models’ reliability based on their consistency with prior knowledge before we have solid theories to endorse them, providing a valuable adjunct to current benchmarking practices. Interpretability can also give us a sounder basis for believing that a seemingly effective prediction model will generalize to new data sets and does not achieve good results through fitting to data artifacts. Furthermore, a major goal of biological research is often to gain understanding of underlying mechanisms, and not simply to solve an engineering issue.

### 3.5 Allowing standards to evolve with the technology

The challenge model remains the gold standard for rigorous benchmarking, but it requires updating for the era of generative AI. For example, multi-track challenges can explore distinct categories designed to separate the influences of data, model advances, and compute resources on performance, such as through open-weight, API-only, or resource limiting tracks. There is no easy fix, however, and complexity of distinguishing these factors will require an ongoing commitment to an evolving, community-driven assessment ecosystem, where living benchmarks and shared evaluation services are regularly discussed and refreshed to keep ahead of advances in AI methods and new ways of unfairly gaming the assessments. A living benchmark would involve ongoing data generation alongside benchmarking, with a data architecture to timestamp data releases and careful accounting for independence to ensure that new benchmarking data is regularly available for independent assessments of models that may have trained on earlier releases of the benchmark. To limit the leaderboard effect, these systems could restrict submission frequency, report rounded or thresholded scores, and separate a public development leaderboard from periodically refreshed, sequestered evaluation sets used for infrequent assessment. Incorporating reusable-holdout methods (Blum and Hardt 2015, Dwork et al. 2015) and continuously adding new data would further reduce adaptive overfitting while allowing benchmarks to evolve as models, datasets, and scientific questions change. In an ideal case, benchmarking might be integrated with data generation in a process of active learning, with new experiments planned to probe most effectively areas of uncertainty of the current models and thus evolve with the challenges of the field. In this way, benchmarking can become a more effective driver of novel advances and mitigate the leaderboard effect that can lead to models excessively tuned to popular benchmarks. Doing so could be accomplished by a significant commitment to novel data generation as part of an existing ongoing challenge, such as CASP, or, conversely, rethinking major data generation efforts to incorporate continuous benchmarking of the tools those data are meant to feed and corresponding course correction of experimental plans based on the results of the benchmarking.

### 3.6 Putting modern AI on a firmer theoretical footing

We can anticipate these standards evolving as the theoretical foundations of modern AI develop, whether that comes from developing frameworks to provide better theoretical guarantees for the currently prevailing deep learning models or from replacing deep learning with other model types that lend themselves better to more efficient algorithms and more rigorous performance guarantees. Theoretical and empirical benchmarking will each have a role to play in assessing improvements in model design or wholly new ideas in modeling and comparing them to prior art. However, before we have answers we need to ask the right questions. What formal computational problem are we actually solving when we train an AI model? By what criteria are we testing that model relative to alternatives and with reference to a benchmark data set?

These are more subtle questions than might be apparent at first glance. The history of theoretical computer science teaches us that formulating the problems correctly can have enormous impact on how they are solved. When we have more rigorous answers to these questions, then we can begin to answer such follow-up questions as whether a method gives formal guarantees of performance, whether one method is provably better than another under a given set of criteria and assumptions, or whether a given method is theoretically optimal for a given problem statement. A focus on more rigorously posing questions of assessment is a first step towards answering those questions more rigorously.

### 3.7 Engaging the community

Bringing better benchmarking standards into practice will require organized support and community engagement beyond what can be accomplished by individual researchers. There is a necessary role for funders in recognizing that sound benchmarking is needed for progress in the field and that this requires support, including committing multi-year support to maintain living benchmarks. Expectations are also needed for sound benchmarking as a condition of consideration for funding, although with the caveats that benchmarking artifacts must be sufficiently lightweight to be accessible to small labs and not just major corporations and that funding will continue to be needed for new directions in research before they have reached the stage where community benchmarks become possible. Similar expectations are needed in other venues, such as publications, to adopt best practices for sound benchmarking of AI methods with similar caveats about the need to ensure that innovations from small labs are not priced out of challenge and that a focus on empirical benchmarks does not block work on innovative new approaches that might need time to compete with more conventional but highly engineered alternatives. The importance and growing complexity of benchmarking suggests a need for ongoing stewardship by neutral authorities; the International Society for Computational Biology or other relevant professional societies might be best positioned to meet this need. Efforts at organizing within the critical assessment community, such as the recently launched Benchmarking, Assessment, and Evaluation Consortium for Science (BEACON: The Benchmarking, Evaluation, and Assessment Consortium for Science 2026) provide a model for such community engagement.

## 4 Conclusions and discussion

As computational tools have become critical to biological discovery, our ability to assess such tools and recognize advances in them has become essential to the practice of rigorous science, whether for the individual user deciding which tools to use, a reviewer or editor assessing significance of advances under review, or a funding agency deciding which research directions will make best use of limited funds. The age of AI has created profound new challenges to the infrastructure of assessment. As we saw above, modern AI poses difficult new problems in scale and character for rigorously assessing methods, comparing them to one another, and judging their likely impact and future promise. Nevertheless, past experience offers some guidance for how we might revise our best practices to give clear guidance to method developers and users. This paper has sought to lay out some of the major challenges and provide potential solutions or at least a path to them.

There are substantial risks to mishandling this issue and, conversely, great opportunities if it is handled well. Biological research today is a data science and the research enterprise depends on computational tools to manage and interpret data. The risk of assessing poorly is that the field will end up using suboptimal tools that lead to suboptimal results—missed discoveries from false negatives and effort wasted on failed validations due to false positives. Even a small difference in effectiveness of a tool for a foundational task, like predicting a regulatory interaction or a functional mutation, might correspond to massive waste in researcher effort and funding by groups that follow up predictions that do not pan out, delaying the path to true discoveries. Even a well-meaning and knowledgeable user today might wade through numerous tools for basic bioinformatic tasks with no sound basis for determining which are truly state-of-the-art and which do not work at all. Poor assessment can also result in the community investing time and limited resources in less promising directions with an opportunity cost to numerous follow-on projects. It can mean failing to invest in potentially groundbreaking technologies because we have no way to distinguish them from the crowd. The opportunities to be gained by more rigorous assessment of AI-driven methodological advances include better finding groundbreaking advances no matter where they originate, targeting them for visibility, supporting their continued development, and bringing them to the applications that will benefit from them.

We close with a consideration of some broader issues for the community in moving forward on this issue. It is a challenge not just to develop guidelines but to bring them into practice in the operation of bioinformatics tool developers, labs using bioinformatics tools, journal editors and reviewers, and funding agencies and their reviewers. The field will also need to grapple with the question of how we will know when AI methods warrant trust comparable to what we might place in a human expert and whether that might eventually displace the need for explainability i.e. often expected today by users of AI in biomedical applications. Furthermore, the technology behind AI continues to evolve in ways that are difficult to predict. For example, quantum computing paradigms (Bose et al. 2026) have potentially substantial advantages over conventional computing in AI, including in the life sciences (Caro et al. 2022) and may offer novel solutions to other challenges mentioned above by promoting model compactness and efficiency, mitigating inductive bias (Gan et al. 2023) allowing for substantially reduced parameter spaces. Other alternatives to conventional computing platforms, perhaps yet to be conceived, could similarly offer both complications and possible solutions as benchmarking continues to evolve with the technologies it assesses. The rise of agentic AI and co-scientists creates new challenges of how to design rigorous quantitative assessments of methods whose goal is complex scientific reasoning rather than more straightforward prediction or classification tasks. Projects like community benchmarks that depend on openness and data sharing are also likely to have to grapple with legal restrictions on data sharing and collaboration that are beyond scientists’ control. These problems also may exacerbate disparities due to differences in infrastructures available to computational juggernauts versus, e.g. academic competitors in developing countries. Finally, adopting our recommendations will require more than just computational biologists following a list of best practices; developers and users of computational biology tools will need to be sufficiently educated in the foundations of modern AI and its uses in biology to apply those standards and verify that they have been applied. That, in turn, requires thinking about how we will update our standards for educating future life scientists who need to make decisions about deploying computational tools to include competence in these topics (Welch et al. 2014, Brooksbank et al. 2024).

## Data Availability

There is no data associated with this manuscript.
